# Glucose-6-Phosphate Dehydrogenase Regulation in Anoxia Tolerance of the Freshwater Crayfish *Orconectes virilis*

**DOI:** 10.4061/2011/524906

**Published:** 2011-10-17

**Authors:** Benjamin Lant, Kenneth B. Storey

**Affiliations:** Institute of Biochemistry and Department of Biology, Carleton University, 1125 Colonel By Drive, Ottawa, ON, Canada K1S 5B6

## Abstract

Glucose-6-phosphate dehydrogenase (G6PDH), the enzyme which catalyzes the rate determining step of the pentose phosphate pathway (PPP), controls the production of nucleotide precursor molecules (R5P) and powerful reducing molecules (NADPH) that support multiple biosynthetic functions, including antioxidant defense. G6PDH from hepatopancreas of the freshwater crayfish (*Orconectes virilis*) showed distinct kinetic changes in response to 20 h anoxic exposure. *K*_*m*_ values for both substrates decreased significantly in anoxic crayfish; *K*_*m*_ NADP^+^ dropped from 0.015 ± 0.008 mM to 0.012 ± 0.008 mM, and *K*_*m*_ G6P decreased from 0.13 ± 0.02 mM to 0.08 ± 0.007 mM. Two lines of evidence indicate that the mechanism involved is reversible phosphorylation. *In vitro* incubations that stimulated protein kinase or protein phosphatase action mimicked the effects on anoxia on *K*_*m*_ values, whereas DEAE-Sephadex chromatography showed the presence of two enzyme forms (low- and high-phosphate) whose proportions changed during anoxia. Incubation studies implicated protein kinase A and G in mediating the anoxia-responsive changes in G6PDH kinetic properties. In addition, the amount of G6PDH protein (measured by immunoblotting) increased by ∼60% in anoxic hepatopancreas. Anoxia-induced phosphorylation of G6PDH could contribute to modifying carbon flow through the PPP under anoxic conditions, potentially maintaining NADPH supply for antioxidant defense during prolonged anoxia-induced hypometabolism.

## 1. Introduction

Glucose-6-phosphate dehydrogenase (G6PDH) is the rate determining enzyme of the pentose phosphate pathway (PPP). It plays an important role in regulating the production of reduced nicotinamide adenine dinucleotide phosphate (NADPH) for many types of biosyntheses as well as pentose phosphates for DNA/RNA synthesis and 3–7 carbon sugars or sugar phosphates for many other uses [1, 2]. One important use of NADPH generated by the PPP is in antioxidant defense, with the NADPH being used to generate the reduced glutathione and thioredoxin that are primary sources of reducing power for antioxidant reactions. Indeed, elevated PPP activity (as a consequence of enhanced G6PDH activity) is often seen under conditions of oxidative stress [3], and G6PDH regulation appears to be critical to antioxidant defense, as seen in multiple studies where G6PDH is disrupted [4, 5]. Regulation of G6PDH has been documented in various systems from bacteria and yeast to plants and animals [6–9] with controls at multiple levels (transcriptional, translational and post-translational), especially under oxidative or salt stresses.

Freshwater crayfish (*Orconectes virilis*) reside in the shallows of streams and lakes, and in this environment are susceptible to low oxygen stress. In the summer, high temperature and low flow conditions can result in hypoxic conditions in the water. In the winter, hypoxic (or even anoxic) conditions can be encountered in ice-locked bodies of water when oxygen is depleted over time by organismal respiration and the inability of photosynthetic plants to replenish oxygen. Hence, crayfish can require good hypoxia/anoxia tolerance and, indeed, several reviews summarize the physical [10] and molecular [11] adaptations used by crustaceans to adapt to hypoxia. Two aspects of hypoxia/anoxia tolerance that have recently received considerable attention are metabolic rate depression (MRD) and antioxidant defense [12–14]. In response to oxygen limitation, hypoxia/anoxia-tolerant organisms strongly suppress the rates of both catabolic and anabolic pathways in a coordinated manner, reducing ATP expenditures over prolonged periods when ATP production by mitochondrial oxygen-dependent mechanisms is interrupted. The main mechanism involved is reversible protein phosphorylation of regulatory enzymes. Several enzymes of carbohydrate catabolism are known targets [12, 13], and recent studies of G6PDH in two natural systems of MRD indicate that control of the PPP under stress conditions is also achieved in this manner. This includes G6PDH from liver of a freeze-tolerant frog (freezing is an anoxic/ischemic stress) and G6PDH from hepatopancreas of an estivating snail (a state of aerobic MRD), both of which showed stress-responsive changes in enzyme properties that were linked with changes in the phosphorylation state of the enzyme [15, 16]. 

Animals that transition into hypometabolic states to survive environmental stress conditions also typically show well-developed antioxidant defenses [14]. These contribute both to long term cell preservation in the hypometabolic state and to dealing with a rapid increase in reactive oxygen species (ROS) production when organisms transition back to oxygenated conditions. This implicates G6PDH as an important enzyme in anoxia tolerance. In the present report we examine the regulation of G6PDH from *O. virilis *hepatopancreas. Relatively few metabolic studies have been done on this invertebrate model but previous experiments revealed regulation of critical protein kinases involved in signal transduction as a response to anoxia exposure [17]. The present study shows that under anoxic conditions there is an increase in the amount of G6PDH present in the high phosphate, low *K*_*m*_ enzyme form, implicating a more active enzyme form under anoxia. *In vitro* incubation studies also demonstrated that changes in G6PDH phosphorylation state that could be mediated by cAMP-dependent protein kinase (PKA) or cGMP-dependent protein kinase (PKG). 

## 2. Methods and Materials

### 2.1. Animals

Freshwater crayfish (*Orconectes virilis*) were obtained from the Britannia Bait and Food market in Ottawa, Ontario. They were kept in aerated containers of fresh water in incubators set at 15°C for 7 days before use. Animals were then separated into groups of 10 and placed in individual covered buckets with 15°C water that was either aerated with a bubbler or deoxygenated by bubbling with 100% nitrogen gas (bubbling with nitrogen gas was begun 45 minutes prior to adding animals). Control animals were sampled from their buckets after 1 hour. Animals in deoxygenated water were sampled after 20 hours of anoxia exposure. Animals were killed by severing the head, and then hepatopancreas was rapidly excised, immediately frozen in liquid nitrogen, and stored at −70°C until use.

### 2.2. Sample Preparation

Typical homogenates were made 1 : 5 w : v in ice-cold homogenization buffer A: 50 mM imidazole, pH 7.0, 2 mM EDTA, 2 mM EGTA, 10 mM *β*-mercapthoethanol, 10 mM *β*-glycerol phosphate, 10% v : v glycerol, and 50 mg phenylmethylsulfonyl fluoride (PMSF) protease inhibitor. Homogenates were centrifuged at 12,000 g for 30 minutes at 4°C. Supernatants were collected and assayed for activity.

### 2.3. G6PDH Activity Assay

Optimal conditions for G6PDH activity were determined to be 0.5 mM NADP^+^ and 5 mM G6P in 50 mM imidazole buffer, pH 7.0; standard assays assayed 20 *μ*L of crude extract in a total of 200 *μ*L reaction mix. Preliminary studies showed that the optimal pH for G6PDH activity was 7.0, added Mg^2+^ did not alter activity, and prior desalting of extracts via low speed centrifugation through small columns of Sephadex G25 (equilibrated in homogenization buffer at pH 7.0) did not affect activity. NADPH production was measured at 340 nm on an MR5000 microplate reader. One unit is defined as the amount of enzyme that utilized 1 *μ*mol of NADP^+^ per min at 22°C. For *K*_*m*_ determinations, each substrate was varied over a range of concentrations (0.1–5 mM for G6P; 0.025–0.7 mM for NADP^+^) at constant cosubstrate concentration. Protein content of the crude extracts was measured using the Coomassie blue dye-binding method with the Bio-Rad prepared reagent. All data were collected using Biolinx 2.0 software and analyzed with MPA and Kinetics 3.51 software [18, 19]. Statistical analysis used the Student's *t*-test or one-way ANOVA with a post-hoc Dunnett's test.

### 2.4. *In Vitro* Incubations to Stimulate Endogenous Protein Kinases and Phosphatases

To determine the effects of endogenous protein kinases and phosphatases on G6PDH activity, samples of crude enzyme extract were incubated *in vitro* with various additives that stimulated the activities of different kinases or phosphatases. Homogenates were prepared 1 : 2 w : v in basic incubation buffer: 50 mM imidazole buffer, pH 7.0, containing 100 mM sucrose. Prior testing showed that activity was stable in this buffer for at least 2 hours. Aliquots of enzyme extract were incubated for 2 h at 4°C as described below. After incubation, samples were centrifuged and then *K*_*m*_ values for NADP^+^ and G6P were measured.

To promote the activities of endogenous protein kinases, incubations contained 5 mM ATP and 30 mM NaF with additions as follows: (a) for PKA: 1 mM cAMP, 10 mM MgCl_2_, and 5 mM Na_3_VO_4_; (b) for protein kinase G (PKC): 1 mM cGMP, 5 mM Na_3_VO_4_, and 10 mM MgCl_2_. 

To promote the activities of endogenous protein phosphatases, incubations contained: (a) for protein phosphatase 1 (PP1): 5 mM Na_3_VO_4_, 2 mM EDTA, 2 mM EGTA, and 2.5 nM okadaic acid; (b) for PP1 + protein phosphatase 2 A (PP2A): 30 mM Na_3_VO_4_, 2 mM EDTA, and 2 mM EGTA; and (c) for PP2B: 5 mM Na_3_VO_4_, 2 mM EDTA, 5 mM CaCl_2_, and 1 *μ*M okadaic acid. A fourth incubation contained basic incubation buffer plus 1 unit of calf intestine alkaline phosphatase. 

For comparison, an “untreated” condition was run, with homogenates prepared in a 1 : 2 w : v in homogenization buffer A, incubated for 2 h at 4°C. After incubation, *K*_*m*_ values for NADP^+^ and G6P were measured.

### 2.5. G6PDH Elution Profiling

Hepatopancreas samples were homogenized 1 : 5 w : v in homogenization buffer B: 50 mM Tris-HCl, pH 9.0 plus all nonbuffer components contained in buffer A. After centrifugation, 0.5 mL aliquots of supernatant were applied to DEAE^+^ Sephadex G50 columns (7.5 cm × 0.75 cm) equilibrated in buffer B. Columns were washed with buffer B and then G6PDH was eluted with a 0–0.5 M KCl gradient in buffer B. Sixty fractions of 10 drops/tube were collected with a Gilson FC203B fraction collector, and 100 *μ*L samples were assayed for activity.

### 2.6. Western Blotting

Frozen tissue samples were crushed under liquid nitrogen and then homogenized 1 : 5 w : v in homogenizing buffer C (20 mM Hepes, pH 7.5, 200 mM NaCl, 0.1 mM EDTA, 10 mM NaF, 1 mM Na_3_VO_4_, 10 mM *β*-glycerophosphate) with a few crystals of PMSF and 1 *μ*L protease inhibitor cocktail added (Sigma-Aldrich, Oakville, ON, CA). Samples were centrifuged as above for 15 min and supernatant was collected. Soluble protein concentration was measured using the Coomassie Blue dye-binding method with a prepared reagent (Bio-Rad, Hercules, CA, USA) and bovine serum albumin as the standard. Sample concentrations were adjusted to a constant 10 *μ*g/*μ*L by the further addition of small amounts of homogenizing buffer and then aliquots of samples were mixed 1 : 1 v : v with 2 × SDS buffer (100 mM Tris-base, 4% w/v SDS, 20% v/v glycerol, 0.2% w/v bromophenol blue, 10% v/v 2-mercaptoethanol) to give a final protein concentration of 5 *μ*g/*μ*L. Samples were boiled for 5 min and chilled on ice prior to loading onto gels.

Aliquots containing 25 *μ*g protein were loaded into wells of 10% polyacrylamide gels, together with prestained molecular weight standards (Bio-Rad) and separated using a discontinuous buffer system [20]. Samples from all experimental conditions were always run on the same gel. Gels were run in a Mini-Protean III apparatus (Bio-Rad) at constant 180 V for 45 minutes at room temperature (RT). Proteins were transferred onto PVDF membranes at 60 V for 90 minutes at 4°C. The resulting blots were probed overnight with primary antibodies for G6PDH (Santa Cruz biotechnology, Santa Cruz, CA, USA—rabbit polyclonal IgG sc-67394) diluted 1 : 1000 dilution in Tris Buffered Saline and Tween 20 (TBST). Blots were then washed multiple times with TBST and incubated with HRP-linked goat anti-rabbit IgG (diluted 1 : 4000 in TBST) (BioShop, Burlington, ON, CA) for 1 hour at RT. Blots were then washed with TBST and developed using enhanced chemiluminescence reagents. Subsequently, blots were stained with Coomassie blue stain (0.25% w/v brilliant blue *R* or a spatula tip full, 7.5% (v/v) acetic acid, 50% (v/v) methanol and 42.5% ddH_2_O) for 10 minutes and destained with destain solution (10% v : v Acetic acid, 30% v : v methanol, and 60% v : v ddH_2_O), for a further 10 minutes.

Bands were scanned using a ChemiGenius Bio-Imaging system, and densitometric analysis was performed using the associated GeneTools software (Syngene, Frederick, MD). To control for irregularities in loading, the chemiluminescent band intensity in each lane was normalized against the corresponding density of one control identified Coomassie stained protein band in the same lane, that was constant between control and experimental conditions (these bands were well separated from the G6PDH band). Mean normalized band densities ± SEM were then calculated; statistical analysis used the Student's *t*-test.

## 3. Results and Discussion

### 3.1. The Pentose Phosphate Pathway under Anoxic Conditions

All organisms respond to hypoxia/anoxia conditions by shifting metabolic fuel use to a high (or even total) reliance on carbohydrate fuels and the glycolytic pathway for generating ATP. Species that have a well-developed anoxia tolerance typically couple this with a strong overall MRD so that ATP demand is lowered to a level that can be supported over the long term by glycolysis alone [13]. G6P sits at an important locus in carbohydrate metabolism and strong enzymatic controls determine its fate in response to different metabolic demands; G6P is produced from both glucose and glycogen and can be utilized in numerous ways including by glycolysis and the PPP as well as for reconversion to either glucose or glycogen. Prominent controls on glycolytic regulatory enzymes via reversible phosphorylation are a common feature of anoxia tolerance in many species but much less is known about regulation of the PPP in response to anoxia and/or during MRD. Estivation-induced phosphorylation of G6PDH occurred in hepatopancreas of the land snail, *Otala lactea, *with effects that would activate the enzyme and it was proposed that this may enhance relative carbon flow through the PPP, compared with glycolysis, during estivation to help maintain NADPH production for antioxidant defense [15]. Analysis of G6PDH from liver of frozen wood found an opposite result—the enzyme from frozen (anoxic) frogs showed a lower affinity for G6PDH substrates that, along with other changes, argued for a reduced role of G6PDH in the frozen animal. The present study evaluates G6PDH regulation with respect to anoxia tolerance in crayfish hepatopancreas. G6P levels typically increase under anoxia, coincident with a switch to reliance on glycogen or glucose as the fuel. In crayfish tail muscle, for example, G6P levels rose from 84 ± 30 nmol/gram wet weight (gww) to a maximum of 535 ± 152 nmol/gww after 4 h anoxia exposure and remained high at 158 ± 77 nmol/gww after 12 hours of anoxia (T. A. Churchill and K. B. Storey, unpublished data). With a higher available substrate concentration, differential regulation of G6PDH under anoxia could be required to regulate flux through glycolysis versus the PPP and serve the needs for antioxidant defense during hypometabolism.

### 3.2. Regulating G6PDH Kinetics through Reversible Phosphorylation

The invertebrate hepatopancreas conducts the functions of the liver and pancreas organs of vertebrates. The organ has an important role in balancing anabolism/catabolism of fuels (including carbohydrates) and also has important roles in antioxidant defense and detoxification. All of these roles can include a need for G6PDH regulation, both in terms of controlling G6P use by catabolic versus anabolic pathways, and in the generation of NADPH reducing power for multiple types of biosynthesis as well as for antioxidant defense. 

**Figure 1:**
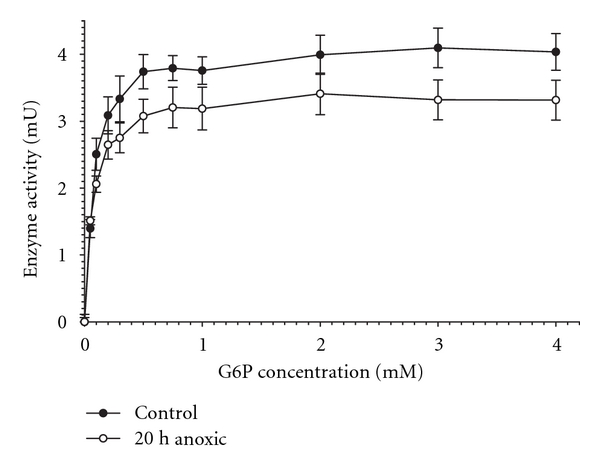
Velocity versus G6P substrate concentration for G6PDH in crude extracts of hepatopancreas from control and 20 h anoxic *O. virilis*. Data are means ± SEM, *n* = 9.

The maximum activity of crayfish hepatopancreas G6PDH did not change between aerobic control and anoxic states (data not shown) but affinity for G6P substrate increased significantly (*K*_*m*_ decreased) between the two states (Table 1, Figure 1).

**Figure 2:**
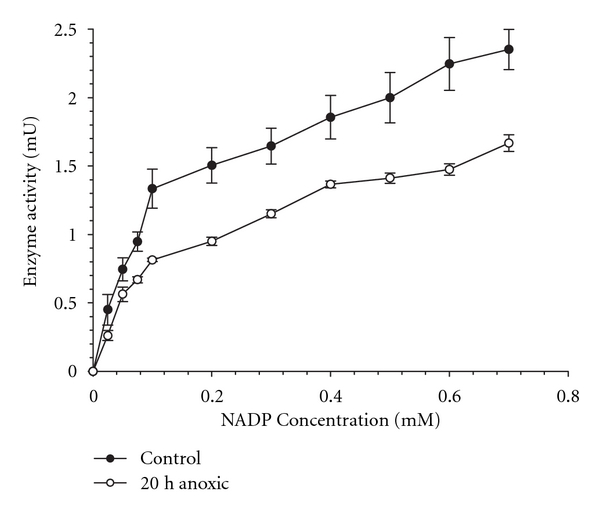
Velocity versus NADP^+^ substrate concentration for G6PDH in crude extracts of hepatopancreas from control and 20 h anoxic *O. virilis*. Data are means ± SEM, *n* = 9.

The *K*_*m*_ G6P fell from 0.13 mM for the aerobic control enzyme to 0.08 mM for the 20 h anoxic enzyme, a 60% decrease for the hepatopancreas enzyme from anoxic crayfish. The *K*_*m*_ value for NADP^+^ also showed a decreasing trend under anoxia as compared with the aerobic control value (Table 2, Figure 2). 

Hence, it appears that the affinity of G6PDH for its substrates is increased under anoxic conditions. These responses to anoxia are similar to the results for G6PDH in other invertebrate models that also show modified G6PDH kinetic properties in response to environmental stress. 

Properties of G6PDH changed significantly between aroused and estivating states in hepatopancreas from the land snail, *O. lactea*; for example, *K*_*m*_ G6P decreased by 50% during estivation [15]. In a vertebrate model, the wood frog *R. sylvatica*, liver G6PDH also showed stress-responsive changes but in this case *K*_*m*_ values for both substrates increased by over 20% during whole body freezing [16]. 

**Figure 3:**
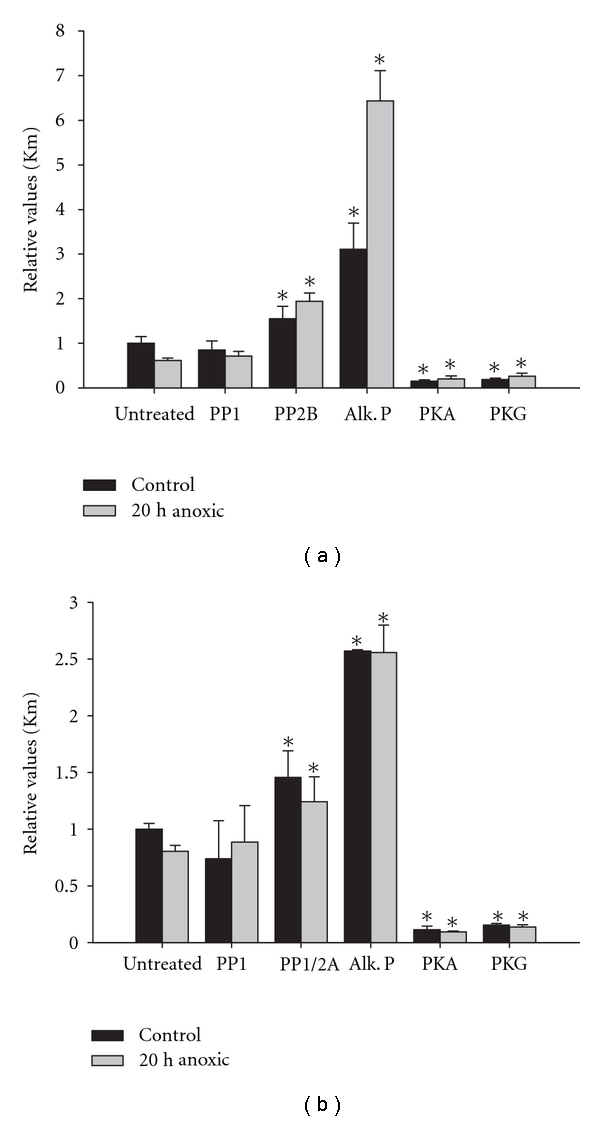
Effects of *in vitro* incubations that stimulated the activities of protein kinases or protein phosphatases on the relative values for *K*_*m*_ for (a) G6P and (b) NADP^+^ for hepatopancreas G6PDH. Data were normalized against the untreated control value that was set to 1.0; values are means ± SEM, *n* ≥ 6 independent. *Significantly different from the corresponding untreated control condition, *P* < 0.05. Note: Kinases and phosphatases stimulated were endogenous, with the exception of alkaline phosphatase.

Stress-responsive stable changes in the properties of enzymes are often due to covalent modification of the protein and frequently these are due to the addition (via protein kinases) or removal (via protein phosphatases) of covalently bound phosphate to the enzyme [12, 13]. To assess whether reversible phosphorylation could also be responsible for the anoxia-induced changes in kinetic properties of crayfish hepatopancreas G6PDH, crude extracts of crayfish hepatopancreas were incubated under conditions that would stimulate the action of selected endogenous protein kinases or protein phosphatases and then changes in the *K*_*m*_ for G6P and NADP^+^ were quantified (Tables 1 and 2) with relative changes in *K*_*m*_ values, compared to aerobic control conditions, shown in Figure 3.

These experiments indicated that the phosphorylation state of G6PDH can be a decisive factor in substrate affinity. Incubation under conditions that promoted the action of PP1 did not affect *K*_*m*_ values but stimulation of PP2 enzymes led to significant increases in *K*_*m*_ for both substrates. Stimulation of PP2B increased *K*_*m*_ G6P of the aerobic control enzyme by 1.5-fold and *K*_*m*_ of the anoxic enzyme by 3-fold. Treatment with commercial alkaline phosphatase produced very large 2.5–6-fold increases in the *K*_*m*_ values for both substrates (Figure 3). Conversely, stimulation of endogenous kinases (PKA, PKG) significantly decreased *K*_*m*_ G6PDH to 15–18% of the untreated control value or to 33–42% of the anoxic value (Table 1, Figure 3(a)). Protein kinase treatments resulted in similar strong decreases in *K*_*m*_ NADP^+^ (Table 2, Figure 3(b)). These data provide strong evidence that the anoxia-induced decrease in *K*_*m*_ values, providing increased substrate affinity, is mediated by protein phosphorylation events; conversely, the return to oxygenated conditions would be mediated by G6PDH dephosphorylation by protein phosphatases. Not only does this identify protein kinases as the activating agent for G6PDH under anoxia but a strong increase in the percentage of PKA present as the free catalytic subunit was found in crayfish tissues under oxygen-limited conditions [17]. It is evident that PKA may be the anoxia-responsive protein kinase that regulates G6PDH *in vivo *but a role for PKG in mediating responses to anoxia cannot be discounted. Indeed, both PKA and PKG treatments altered G6PDH activity and elution profiles in hepatopancreas extracts from *O. lactea* [15].

### 3.3. DEAE Ion Exchange Separation of G6PDH Forms

**Figure 4:**
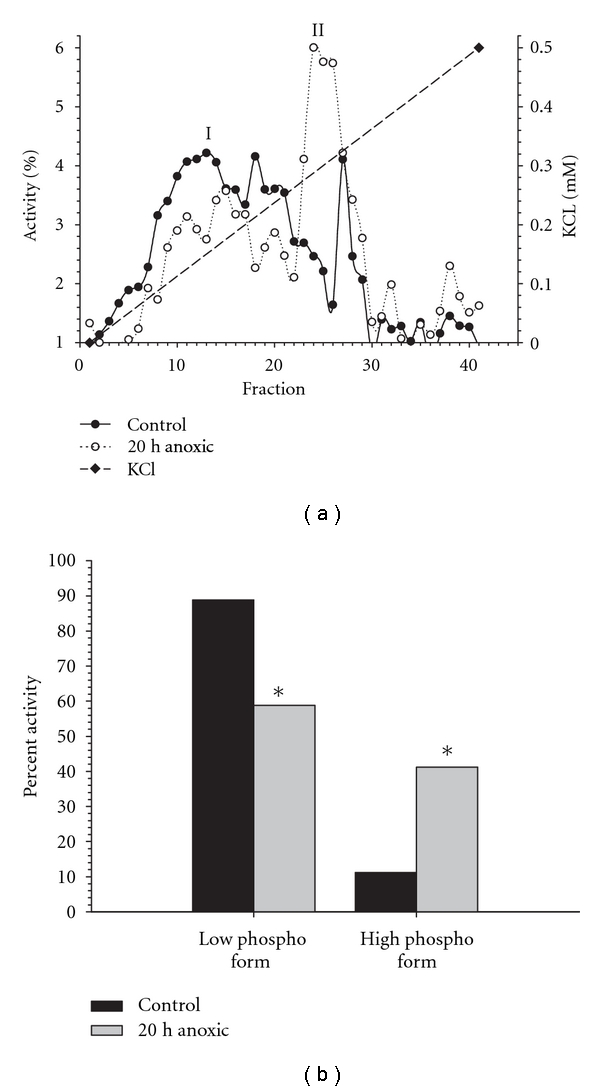
(a). DEAE-G50 Sephadex elution profile for G6PDH from hepatopancreas of control and 20 h anoxic *O. virilis*. The elution profile is representative of three independent determinations for both control and anoxic conditions. Activity is the percentage of activity retrieved from the column. (b). Percentage of G6PDH activity in the low (Peak I) and high (Peak II) phosphorylated forms in *O. virilis* hepatopancreas under aerobic control and anoxic conditions.

Changes in the phosphorylation state of a protein alter its charge and hence the high and low phosphate forms of enzymes are typically separable on an ion exchange column.  Figure 4 shows the elution pattern of hepatopancreas G6PDH from aerobic and anoxic crayfish on DEAE ion exchange chromatography. Activity eluted in two main peaks and the ratio of the two peaks changed between the two physiological states. Under aerobic conditions, the majority of the activity (88.8% of recovered activity) eluted in a broad peak at a low KCl concentration (Figure 4(a), peak I). Under anoxia, however, the percentage of activity in peak I decreased to 58.8% of recovered activity with a second large peak of activity eluting at a higher salt concentration (Figure 4(a), peak II). The higher the content of bound phosphate on a protein, the greater its negative charge and the more tightly it should bind to DEAE; therefore, a higher concentration of KCl should be required for elution. Hence, peaks I and II would represent the low- and high-phosphate forms of G6PDH, respectively. 

This also agrees with the outcome of the kinase and phosphatase incubations. Anoxia exposure reduced the *K*_*m*_ values for both substrates. This was mimicked by stimulating protein kinase actions on the enzyme; both PKA and PKG action on G6PDH strongly decreased the *K*_*m*_ values for both substrates (mimicking the effects of anoxia) indicating that the anoxic enzyme is the high phosphate form. By contrast, incubation of the anoxic enzyme formed under conditions that stimulated PP2 action raised *K*_*m*_ values to those that mimicked or exceeded the control values, indicating that G6PDH in hepatopancreas of aerobic crayfish is the low phosphate form. The elution profiles also show that under both aerobic and anoxic conditions that G6PDH exists in two forms—both high and low phosphate forms are present and anoxia exposure shifts the proportions of the enzyme in each form. While the elution profile does not indicate a target kinase, it does confirm a phosphorylation event during anoxia. Hence, *K*_*m*_ values for the untreated control and anoxic forms represent the value for a mixture of the two forms. The incubation treatments with PKA/PKG versus alkaline phosphatase indicate that there is a much wider scope for change in *K*_*m*_ if the enzyme is fully phosphorylated or dephosphorylated. For example, *K*_*m*_ G6P values ranged from 0.019 to 0.034 mM when fully phosphorylated to 0.40–0.83 mM when fully dephosphorylated, giving an approximately 10–40-fold scope for change in *K*_*m*_ G6P. Similarly, *K*_*m*_ NADP was 0.014–0.023 after PKA/PKG treatment and 0.38 mM after alkaline phosphatase treatment, a 16–27-fold difference between fully phosphorylated and fully dephosphorylated enzyme forms.

### 3.4. Protein Levels of G6PDH Increase under Anoxia

Along with kinetic changes regulated by posttranslation modification, changes in the total amount of G6PDH protein could also be a factor in G6PDH control under anoxia. There is a precedence for stress-mediated G6PDH expression level changes: both RNA and protein. Under salt stress in reed callus [8] and wheat [21], G6PDH expression levels are distinctly responsive. In both cases NaCl treatments caused marked increases in G6PDH protein levels [8] and RNA levels [21]—particularly in longer (>12 h) exposures. 

**Figure 5:**
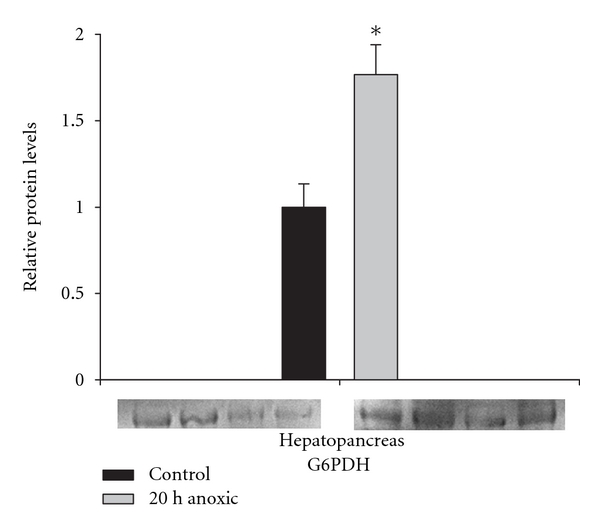
Effect of 20 h anoxia exposure on the protein levels of G6PDH in *O. virilis* hepatopancreas, as determined by Western blotting. Immunoblot bands for *n* = 4 independent samples are shown, and histogram shows normalized protein levels. Data are means ± SEM, *n* = 4. *Significantly different from the corresponding control, *P* < 0.05.

G6PDH protein levels were analyzed by Western blotting, the results showing a 1.6-fold increase in total protein levels in *O. virilis* hepatopancreas under anoxia (Figure 5). 

This suggests a twofold mechanism for enhancing G6PDH activity in hepatopancreas under anoxia—an increase in G6PDH protein levels and reversible phosphorylation mediated conversion of the enzyme to a more active form with enhanced substrate affinity. This dual action mechanism supplies strong evidence that G6PDH regulation is important to anoxia survival and/or aerobic recovery from anoxia, potentially by modifying G6P flux though the PPP under anoxia and providing enhanced NAPDH availability for antioxidant defense.

This follows the trend established among other stress-tolerant animals: G6PDH from *O. virilis* sharing the invertebrate protein expression pattern of increasing under stress conditions. Specifically, the land snail (*O. lactea*) G6PDH shows increased peak II (high phospho) protein activity, which is also less susceptible to urea degradation [15]. Similarly, *O. virilis* G6PDH protein expression differs from the vertebrate model (*R. sylvatica*), which appears not to change under freezing exposure [16].

## 4. Conclusions

As oxidative stress becomes prolonged, in a stress adapted animal, energetically expensive procedures are limited, while defenses (heat shock proteins, stress activated protein kinases, antioxidant proteins, etc.) are triggered and accumulated. The anoxia-tolerant freshwater crayfish, *Orconectes virilis*, is no exception to this and could consequently experience a reduction in metabolic rate of up to 90% [12], during its overwintering period. The increased protein levels and activity of *O. virilis* G6PDH, which increase substrate affinity of both G6P and NADP, can have a twofold effect; first to divert G6P away from glycolysis and second to generate reducing molecules (NADPH).

The NADPH produced can then be used to return glutathione (GSSG) to its reduced form (GSH) which is the substrate for two powerful antioxidant enzymes, glutathione peroxidase (GPX) and glutathione-*S*-transferase (GST) [22]. NADPH is also used to replenish reduced thioredoxin which contributes to peroxiredoxin antioxidant defenses [22], as well as providing the reducing power for the cytochrome P450 enzymes that mediate detoxification and xenobiotic transformation reactions [23]. Thus, increased NADPH production underlies the majority of antioxidant defenses necessary both for long-term survival in the hypometabolic sate and in preparation for the substantial influx of oxygen at the end of an anoxic excursion. Without these defenses, accumulation of ROS could cause severe cellular damage, as is the case in numerous examples where experimental manipulations have inhibited/reduced G6PDH activity [5, 24, 25].

In summary, these data illustrate a potentially conserved invertebrate mechanism of G6PDH regulation under oxidative stress. Under anoxic conditions, *O. virilis* shows increased G6PDH protein content in hepatopancreas and a stable modification of the enzyme via phosphorylation (possibly mediated by PKA) that increases substrate affinity. This implicates a coordinated molecular response of the crayfish to oxidative stress in order to maintain energetic homeostasis and generate antioxidant defenses in the hypometabolic state.

## Figures and Tables

**Table 1:** Effect of incubation under conditions that stimulated the activities of protein phosphatases or protein kinases on the *K*_*m*_ values (mM) for G6P of crayfish hepatopancreas G6PDH.

Condition	Control *K*_*m*_ (mM)	20 h Anoxic *K*_*m*_ (mM)
*Untreated*	0.13 ± 0.016	0.08 ± 0.007^b^
*PP1*	0.11 ± 0.026	0.092 ± 0.014
*PP2B*	0.20 ± 0.036^a^	0.25 ± 0.025^a^
*Alkaline Phosphatase*	0.40 ± 0.076^a^	0.83 ± 0.088^a,b^
*PKA*	0.019 ± 0.039^a^	0.026 ± 0.009^a^
*PKG*	0.024 ± 0.039^a^	0.034 ± 0.009^a^

Data are means ± SEM, *n* = 3-4. ^a^ Significantly different from the corresponding untreated value, *P* < 0.05; ^b^ significantly different from the corresponding control value, *P* < 0.05.

**Table 2:** Effect of incubation under conditions that stimulated the activities of protein phosphatases or protein kinases on the *K*_*m*_ values (mM) for NADP^+^ of crayfish hepatopancreas.

Condition:	Control *K*_*m*_ (mM)	20 h Anoxic *K*_*m*_ (mM)
*Untreated*	0.15 ± 0.008	0.12 ± 0.008^b^
*PP1*	0.11 ± 0.05	0.13 ± 0.048
*PP1/2A*	0.22 ± 0.035^a^	0.19 ± 0.033
*Alkaline Phosphatase*	0.38 ± 0.002^a^	0.38 ± 0.036^a^
*PKA*	0.017 ± 0.005^a^	0.014 ± 0.001^a^
*PKG*	0.023 ± 0.002^a^	0.020 ± 0.003^a^

Data are means ± SEM, *n* = 3-4. ^a^ Significantly different from the corresponding untreated value as determined using ANOVA and a post hoc Dunnett's test, *P* < 0.05; ^b^ significantly different from the corresponding control value, *P* < 0.05.
